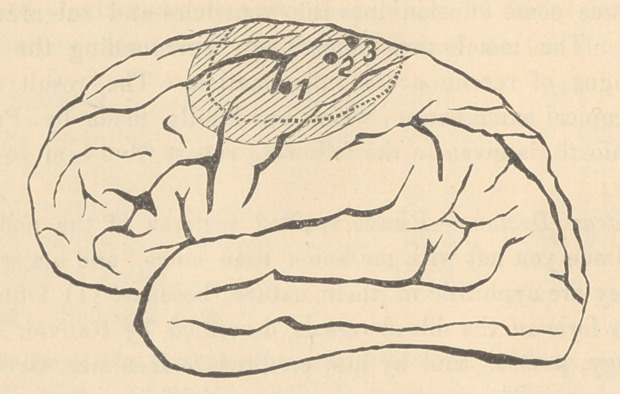# Case of Epileptiform Convulsion and Paralysis, Due to Syphilitic Tumor of the Cortex in Motor Area

**Published:** 1883-01

**Authors:** Franklin H. Martin

**Affiliations:** Assistant


### ARTICLE II.

### CASE OF EPILEPTIFORM CONVULSION AND PARALYSIS DUE TO SYPHILITIC TUMOR OF THE CORTEX IN MOTOR AREA. Case from St. Joseph's Hospital, Nervous Department, PROF. D. R. BROWER, M. D., Physician in charge.

Reported by FRANKLIN H. MARTIN, M.D., Assistant.

The peculiarity and rarity of the following case, together with the completeness of its history and the rare opportunity it afforded for a post-mortem examination, justifies us in taking the considerable space we have for its report.

The patient had his first attack in the latter part of the year 1880. The following February, by advice of his family physician, Dr. Woodworth, he consulted Prof. J. S. Jewell. Through the kindness of Dr. Jewell, I have been permitted to use the following history of the case, taken by him at that time: "February 14, 1881. R. S. P——, age 35, weight 174 pounds. Neuro-sanguineous temperament. Maker of deformity apparatus. Worked in a close room, not well ventilated. Before this time was a traveling man, and had been healthy. Had an injury when young, on the top of the head from a fall. Scalp wound. Never had sunstroke. No rheumatism. May have had syphilis. Has had other genito-urinary diseases. Has had organic stricture of the urethra. Has had considerable alcoholic abuse. Used, and uses tobacco, strong tea and coffee. No medicine habits. No malaria. Appetite good. Good eater and has been a high liver. Digestion only fair. Wind occasionally on stomach. Nausea rare. Costive—has to use laxatives. No piles. No bowel disease. No worms. Urine passes fairly when stricture permits. No pain in passing urine. Tone of bladder fair, and no bladder disease. Strong sexual desire, although thinks he has never over-done in this way.

"At times has pains in heart. 'Faints away,' or latterly 'has fits.' Heart palpitates easily. Hands and feet cool at times. Has rush of blood to head often. No lung disease. Sweats very easily. Has wandering headaches, more severe on top of the head. For a year or so prior to October last, had a node (?) on left upper forehead.

"Vision fair in right eye. The left eye has been blind for twenty years. Not a complete loss of vision. Cannot say whether the left visual field has any play of objects. Does not know the cause of his loss of vision. Hearing good. *Tinnitus aurium* at times. Other special senses in good condition.

"Rather strong, but myasthenic. Not often dizzy, but has at times dizzy spells. Has to cease work at these times and sit down. Co-ordination fair. Dynamometer tests, and tendon reflex fair. Does not sleep well. Dreams much. Bad dreams. Restless. Muscular twitchings. The patient is neurasthenic. Memory fair. Absent-minded. Cerebro-asthenic. Cheerful as a rule. Is irritable and easily annoyed lately.

"Came out of a nervous family. Brother died of nervous disease. Mother died of nervous trouble.

"October last, had a 'fit' at 12 at night; came on while asleep; another at 5 a. m.; unconscious. Next 'fit' January last. This one while in the shop at work. Since then has had several fainting spells. Begins with a staring sensation. There is a play of heat waves in visual field which rise upward—then he has the 'fit.' The pupils are dilated. Difficult breathing. Froths at the mouth, and hands clenched. Does not know about what way head turns, or whether convulsions are symmetrical."

At the time the above history was taken, Dr. Jewell put the patient upon a thorough course of the mixed bromides, and iodides. He was upon this treatment until May 5, 1881, when the following note was made: "Head turns to left during convulsions. Eyes turn away. Passes hand to mouth and works to remove his artificial teeth. Been much better. I advise him to go on with same medicine. He stopped medicine for ten days and had four 'spells.' Had warning of their approach, which gave him time to prepare. Had one lasting 15 minutes (? less). Had another the same night while sleeping. The next evening a light one, and did not lose consciousness."

July 2d, the following note was made: "Has had some slight attacks—six to ten. Begins now with a curious creeping sensation in the left side of the tongue, from tip to one-half distance back, and often, also, at left roof of mouth about the same distance back as on the tongue. Pulse weak. Confused in his head, but conscious, and at times unable to talk, but can think of the words. Aphasia lasts but a few minutes. Convulsions may be caused, by touching the left side of the tongue with a piece of paper, or anything of the kind, as he has done occasionally by accident. At one attack the whole left arm was utterly paralyzed in sensation for one-fourth hour, and weak as to motility. The left cheek feels heavy and dead after convulsions. Left arm yet weak."

At the time the above note was made, July 5th, a slight change was made in the patient's treatment, by adding to his bromide mixture fifteen minim doses of the fluid extract of ergot, and small doses of the tincture of digitalis.

Three days after this, he started for Yarmouth, N. S., where he remained until September, when he went to New York City.

Soon after reaching Yarmouth, he discontinued Dr. Jewell's treatment, and consulted Dr. Hutchins, of that city, who put him upon a bromide and ergot prescription with iodide of potassium. On account of a severe pain in the head that was troubling him at this time, he was given in addition to the above treatment, a prescription containing tincture of opium, tincture of cannabis ind. and gelsemium. After going to New York City in September, he discontinued all drugs, and received "electrical treatment" from some irregular. The next month he left New York and came to Chicago: staid here two weeks, and went to New Orleans. From there he returned the following April. From the time he left Chicago the first time (July 5, 1881), until a short time before he returned, the last of April, 1882, he was very well and able to work—having had but three fits during the whole time. While in New York he had "a stroke of paralysis." It was impossible, under the circumstances, to learn what, and how extensive this attack was. It was doubtless one of his severer convulsive attacks.

After returning from the South, the patient commenced to have frequent attacks of vertigo, accompanied by slight convulsive movements of the muscles of the face and eyeballs. These would come on suddenly while walking in the street, or while working. Sometimes the patient would be able, with a powerful effort of the will, to ward off an attack. The "spells" increased in frequency and severity, until he came under our observation at St. Joseph's Hospital.

The patient at this time, July 13, was able to walk about the ward, and to a certain extent wait upon himself. The convulsions came on at irregular intervals of fifteen minutes to an hour, and they would vary in length from thirty seconds to a couple of minutes. The patient retained consciousness during the convulsions to a remarkable degree. He would demonstrate this feature by remembering and commenting upon remarks that were made by the attendant during his attack. By irritating the conjunctiva, he would give signs of sensibility, and afterward ask why it had been done.

The "fit" would begin by the patient experiencing a twitching of the eyes, and oppression of the chest, by which he would realize at once that the couvulsions were about to appear. Clonic contractions of the muscles of the eyeballs, and of the superficial muscles of the left side of the face, would now begin, followed by like contractions of the deeper muscles of the face, soon extending to the muscles of the neck, causing the head to be convulsively drawn to the left side. From here the contractions extended to the muscles of the left shoulder, arm, fore-arm and hand, and finally, in the severer attacks, to all the voluntary muscles of the left side. Respiratory movements were very irregular. The face, which at the beginning of an attack was pale, during the progress of the "fit" became congested, and almost black. Both eyeballs were rolled upward, and to the left, by the powerful clonic contraction of their muscles; the eyelids were convulsively opened and closed, and the whole appearance of the patient was that of one laboring to contract every fiber of every muscle that was in action. The convulsive movements usually passed off suddenly, and the patient would immediately regain composure, and be ready to resume any conversation that might have been interrupted. The powerful contractions caused him much suffering.

After each "fit," the patient complained of powerlessness of the left arm, and as the convulsions increased in severity and number, this became more and more aggravated, and gradually extended to the lower extremities, and finally amounted to complete loss of motion of the muscles of the left side. This paralysis came on gradually during the last two weeks of his illness, and was not complete until about three days before his death. The right side was not affected. Sensibility was not much impaired.

By experimenting with the surface brain thermometer, we observed near the right parietal eminence, extending to the median line, a spot about two inches in diameter, that exhibited a well-marked elevation of temperature. Its outlines were well defined. Moving the thermometer the width of the bulb surface in any direction from this spot would make a difference in temperature of from two-fifths to three-fifths of a degree. With the exception of this particular spot, the temperature of the two sides of the brain was the same. The surface temperature, as taken over this particular portion the last six days before death, and that of the other portion, may be compared in the following record that was taken at the time.

The first column represents the temperature of the right side over the surface mentioned; the second, that of the left side:

| | | |
|---|---|---|
| Friday morning | 98 3-5° | 98° |
| Saturday morning | 98 2-5 | 97 9-10. |
| Sunday morning | 99 1-5 | 98 4-5. |
| Monday morning | 98 2-5 | 98 1-5. |
| Tuesday morning | 98 4-5 | 98 2-5. |
| Wednesday morning | 100 4-5 | 99. |
| Thursday, died at 2 A. M. | | |

The treatment the patient received while with us, was simply the mixed iodides and the bromides in appropriate doses, with the usual accompanying diet list, composed of the simplest nourishing food.

*Post-mortem.*—On inspection, the body exhibited old scars in numerous places, which helped to confirm the syphilitic theory. The superficial lymphatic glands were not apparently much enlarged. The body was well-developed; did not show signs of much emaciation. Upon removing the scalp, the surface of the skull presented nothing unusual. The calvarium was next removed. The inner surface exhibited nothing abnormal, except an unusually large size and number of the Pacchionian bodies. Turning attention to the brain, before disturbing its membranes or its position, the eye was at once attracted by a thick opaque deposit or tumor, about two inches in diameter, situated beneath the dura mater, on the upper surface of the right hemisphere. Its anterior portion occupied the posterior extremities of the first, second and third frontal convolutions; its posterior portion covered the superior extremity of the ascending frontal convolution. Its posterior border overlapped the fissure of Rolando, and its superior or internal border extended to the longitudinal fissure. The dura mater was closely united to the surface of the tumor, to the extent of from perhaps one and a half to two inches square, very much thickened, and of a yellow translucency. In a section made perpendicularly into the tumor through the dura mater, it was seen that the dura mater, the arachnoid, the pia mater and surface of the brain were blended into an unrecognizable mass. There was some effusion into the ventricles and sub-arachnoid spaces. The membranes immediately surrounding the tumor gave signs of recent acute inflammation. The result of the microscopical examination, which was kindly made by Prof. I. N. Danforth, is given in the following report from him to Prof. Brower:

*My dear Doctor:*—I have studied sections of the nodule of brain-tissue you left with me some time since, and am satisfied that they are syphilitic in their nature, because, (1) I find the peculiar form in the blood-vessels described by Ranvier (Path. Histology, p. 351), and by him credited to Heubner, Greenfield and Barlow. This lesion consists essentially in a diminution of the lumen of the vessels, and this is due to two causes; thickening of the inner coat by a cellular growth very much like granulation tissue, or embryonal tissue; and also a thickening (hypertrophy) of the external layer (adventitia) and this is due to a multiplication of spindle cells—in fact, the production of a low form of connective tissue.

(2) I find the characteristic increase of connective tissue cells (cells of the neuroglia) in the indurated nodular masses. The branched connective tissue cells are not present—at least, I have not found them. But that is not an essential point; in fact, it only indicates that the connective tissue cells have grown too rapidly to admit of perfect differentiation.

Yours truly,

I. N. DANFORTH.

It will be noticed that this case is one of the very few actual clinical cases reported that help to confirm Hitzig's and Ferrier's theory of motor localization in the cortex of the brain. Observe the accompanying drawing from Hitzig, with explanations, the dotted tracing giving location of the tumor.

Assuming that that the accompanying cut is correct in every respect, it is easy to explain the various manifestations of the convulsions in the different stages of our case, while the tumor was developing. The tumor commenced as a small nodule, undoubtedly, situated entirely in the membranes, and by its presence, simply irritated the motor centers controlling the muscles of the face for one side, this irritation causing these muscles to contract.

The disease gradually extended in the membranes, irritating successively the motor centers for the upper extremity, the trunk, and finally the lower extremity. Soon the disease invaded not only the membranes, but the tissue of the brain immediately beneath them, thereby destroying the tissues or centers of motion it had heretofore but irritated. Of course, as these centers were invaded and destroyed, exactly to that extent were the muscles that they controlled paralyzed.

## Figures and Tables

**Figure f1:**